# Application of Manuka honey in treatment patients with GERD

**DOI:** 10.1002/fsn3.3748

**Published:** 2023-10-12

**Authors:** Michał Gośliński, Dariusz Nowak, Roman Mindykowski, Wojciech Kulewski, Cezary Popławski

**Affiliations:** ^1^ Department of Nutrition and Dietetics, Faculty of Health Sciences, Ludwik Rydygier Collegium Medicum in Bydgoszcz Nicolaus Copernicus University in Toruń Bydgoszcz Poland; ^2^ Department of Gastrointestinal Endoscopy University Hospital No. 1 in Bydgoszcz Bydgoszcz Poland

**Keywords:** endoscopy, esophagitis, GERD, Manuka honey, nutritional interview

## Abstract

Gastro‐esophageal reflux disease has systematically increase in Western countries over recent years. Health benefits of Manuka honey allows to use it for medical purposes, for example reduction of inflammation of gastrointestinal mucosa. Thus, the aim of research was the application of Manuka honey in treatment patients with Gastro‐esophageal reflux disease (GERD). The study was conducted on a group of 30 patients, which consumed Manuka honey or placebo for a period of 4 weeks. The gastroscopy and histology has been made twice, that is before and after experiment. Furthermore, the symptoms reports and nutritional interviews have been collected. The endoscopical examination showed that in Manuka group the improvement was 73.3%. In the sub‐groups of esophagitis‐A and esophagitis‐B the improvement rate was 81.8%, and 50%, respectively. However, in the placebo group, the general improvement rate was much lower. The results have been confirmed in the histopathological examination. Moreover, it is worth noticing, that in sub‐group declaring non‐medication, the improvement was only for patients consuming Manuka honey. Changes of symptoms in subjective assessment of patients were evaluated after 2 and 4 weeks. The improvement rate in Manuka group was 86.7% and 100%, while in Placebo group it was only 26.7% and 40%, respectively. Manuka honey seems to be effective in GERD, which have been confirmed by subjective feelings of patients and by endoscopic and histopathologic examination. Our research is a pilot study before administration of Manuka honey to larger population. The results are promising and may facilitate the quality of life of patients with GERD.

## Abstract 2

To the best of authors knowledge, there is no other study concerning the use of Manuka honey in gastro‐esophageal reflux disease. Manuka honey seems to be effective in Gastro‐esophageal reflux disease (GERD), which have been confirmed by subjective feelings of patients and by endoscopic and histopathologic examination. Obtained results are very encouraging and may improve treatment of GERD and facilitate the quality of life of patients, for example relieving heartburn and regurgitation. Manuka honey combined with proper diet and drugs could be a triple therapy regimen for GERD.

## 1 INTRODUCTION

Gastro‐esophageal reflux disease (GERD) is one of the most common diseases in world population. The failure of the physiological equilibrium between the protective and aggressive factors in the esophagus and the failure of the lower esophageal sphincter are the main pathological reason of the disease (De Giorgi et al., 2006; Jarosz & Taraszewska, 2008; Vakil et al., 2006).

Over the last several years, there has been a systematic increase in the incidence of GERD, particularly in developed countries (Western Europe, USA, Canada). This applies equally to men and women, and the increasing of symptoms appears above 40 years of age. It has been estimated, that the main symptom of GERD (i.e. heartburn) affects everyday 7%–10% of the population, and at least once a week approximately 10%–20% (Dent et al., 2005; Jarosz & Taraszewska, 2008).

This problem also applies the Polish population (Reguła et al., 2005). There are two types of reflux: physiological, which occurs most often after a hearty meal, and pathological reflux, leading to the damage of esophageal mucosa. It is suggested that the causes of the disease are associated with changes in lifestyle (stress, obesity, physical activity) and because of the way of eating. Typical symptoms of reflux: heartburn, acid regurgitation, painful swallowing, and sometimes nausea. There is also a possibility of some non‐esophageal symptoms, such as laryngological (hoarseness, aphonia), cardiological (chest pain) or pulmonary (cough, shortness of breath, bronchial asthma) (Boeckxstaens, 2007; Jarosz & Taraszewska, 2008; Vakil, 2010; Vakil et al., 2006). Endoscopy is useful diagnostic tool for GERD. The treatment of GERD includes pharmacological and non‐pharmacological therapies. The most useful medications in GERD treatment are the substances decreasing acid contents in stomach juice. Moreover, anti‐secretory medication such as histamine II receptor antagonist or proton pomp inhibitors (PPI) have been used to treat patients suffering GERD (Jarosz & Taraszewska, 2008; Kahrilas et al., 2008). For most people (75%–90%), the symptoms of reflux disease disappear after 6–8 weeks of such treatment. Unfortunately, in a large number of cases they tend to be recurrent. On the other hand, significant number of treated patients still have typical symptoms of GERD such as: heartburn, regurgitation, chest pain and the quality of life (QOL) in that group is lowered. Some of the patients do not develop complete mucosal healing after treatment. The PPI are effective at healing reflux esophagitis, but they are not so effective at relieving GERD symptoms such as heartburn (Jarosz & Taraszewska, 2008; Kahrilas et al., 2008; Vakil et al., 2004). In large multicenter, randomized, double‐blind, parallel group trial esomeprazole and lansoprazole reached healing rate for erosive esophagitis greater than 85% at 8 weeks. Unfortunately, these medications did not perform quite as well with GERD symptoms. At 4 week 40% of patients reported persistence of GERD symptoms (Castell et al., 2002). Chronic gastroesophageal reflux disease leads to complications such as ulceration, bleeding or esophagus narrowing. The most dangerous complication is Barrett's esophagus, which is a precancerous condition (Boerwinkel et al., 2014). Moreover, it is still unclear if PPI can prevent the development of cancer in Barrett's esophagus. Based on that all observation novel therapeutic targets, other that gastric acid production and bile components of refluxate are needed for treating reflux esophagitis and its complications (Spachler et al., 2011). Non‐pharmacological way includes modification of a diet and changes in lifestyle. Easily digestible diet with limitation of substances that stimulate gastric acid secretion is recommended (Akbar & Howden, 2016; Jarosz & Taraszewska, 2014; Meining & Classen, 2000). Many studies show that natural antioxidants supplied with food may play important role in prevention and prophylaxis of certain civilization diseases. These compounds are responsible for free radicals scavenging and cause significantly decrease of oxidative stress (Broncel et al., 2010; Huang et al., 2012). An important role is played by fruit and vegetables, and good quality juices rich in polyphenols. Bioactive compounds, in particular antioxidants, possess cardioprotective, antiinflammatory, antidiabetic, and anticarcinogenic properties (D'archivio et al., 2007; Dauchet et al., 2006; Heiss et al., 2010; Kulling & Rawel, 2008; Nowak et al., 2017). Furthermore, honey is valuable source of ingredients of antimicrobial, antioxidant, and anti‐inflammatory effects (Bogdanov et al., 2008). Current studies show that better properties possess dark honeys (buckwheat, heather, honeydew) than light honeys, and multifloral better than unifloral. Recent data stated that Manuka honey, in comparison to other honeys, is characterized by high content of polyphenols and is relatively high in antioxidant capacity. This product originated from New Zealand or Australia, and is produced by honeybees that collect nectar from Manuka bush (*Leptospermum scoparium*)—*Myrtaceae* family. It has potent bactericidal and bacteriostatic features (Alzahrani et al., 2012; Marshall et al., 2014). This is due to the presence of polyphenols, particularly flavonoids and phenolic acids and methylglyoxal (MGO), which in other honeys is in trace amount (Kwakman et al., 2011; Marshall et al., 2014; Mavric et al., 2008). A strong bactericidal effect of MGO coupled with the content of various bioactive compounds allows to use Manuka honey for medical purposes, for example acceleration of wound healing or reduction of inflammation of gastrointestinal mucosa (reduction gastroenteritis). Manuka honey has been used in supporting the treatment of gastric ulcers, combined with antibiotics (synergy effect)—amoxicillin and clarithromycin (Nzeako & Al‐Namaani, 2006).

Taking into account the unique ingredients and health benefits of Manuka honey, there is an opportunity for its application in a group of patients with upper gastrointestinal tract diseases, such as GERD and gastritis. Thus, the aim of our study was the oral application of Manuka honey in treatment patients with GERD.

## 2 MATERIALS AND METHODS

### 2.1 Subjects

Participants were recruited from the Department of Gastrointestinal Endoscopy of University Hospital no. 1 in Bydgoszcz. The experimental protocol was approved by the Bioethical Committee (KB no 165). All subjects signed an informed consent after reading of the purpose and research schedule. Participants inclusion criteria were: age 18–75 years, both sex, referral from a general practitioner with dyspeptic symptoms or heartburn, recurrence of a previously diagnosed GERD or gastritis, GERD symptoms despite the classical pharmacological treatment (persistent GERD). Exclusion criteria were: antibiotic therapy, chronic inflammatory diseases, chronic organ disorders, diagnosed neoplastic disease, uncontrolled diabetes, documented allergic incidents (in particular bee venom allergy), and alcohol abuse.

The study was conducted on a group of 35 patients (14 men and 21 women) aged between 21 and 75 years (mean 55.1 years). Calculated BMI showed that 54.3% of participants had normal weight (18.5–24.9), 34.3% were overweight (25.0–29.9) and 11.4% class 1 obesity (30.0–34.9). Among subjects, 71.4% lived in the big cities, 14.3% in towns and 14.3% in countryside. Education levels were as follows: 28.6% high school graduates, 57.1% college graduates, 8.6% technical, and 5.7% primary school graduates. Patients also declared how long they struggled with various reflux symptoms: 77.1% indicated the period over 12 months, 5.7% of 3–6 months, and 17.2% up to 3 months. Finally, five people resigned from the survey without giving a particular reason. Participants qualified for the study have been randomly divided into two groups (15 person each—experimental and control group). The selection into two groups was aided by a random number generation formula (an MS Excel application). Research groups were as follows: Manuka group (*n* = 15; 4 male and 11 female; mean 54.9 years) and Placebo group (*n* = 15; 8 male and 7 female; mean 59.1 years).

### 2.2 Manuka honey

Research was conducted using orginal Manuka honey received from Manuka Health New Zealand Ltd. via Propharma Sp. z o.o. (Warsaw, Poland). Every batch of honey was tested and certified for guaranteed quality and methylglyoxal content (at least 400 mg/kg of methylglyoxal).

Manuka honey and placebo (artificial honey) were prepared in the form of ready to use by patients. For this purpose, samples were confectioned into single units of 5 g weight, which ensure that every person receive the same dose of honey. Then, the samples were vacuum packed in blister packs, frozen and stored under refrigerated conditions.

### 2.3 Study protocol

Participants consumed Manuka honey or placebo (artificial honey) in a unit dose of 5 g, three times a day, that is fasting in the morning and between main meals, for a period of 4 weeks.

After the initial selection, we collected blood from participants for biochemical testing (baseline ‐ “zero sample”). This operation was repeated after 2 weeks of consuming Manuka honey, and next after 4 weeks, that is at the end of the experiment.

The gastroscopy has been made in all patients from both experimental and control group. First gastroscopy was made after the initial selection, second after the 4 weeks of experiment. The histology samples from esophagus and stomach were collected during the endoscopy.

Endoscopy examination was made after typical xylocaine anesthesia. All gastroscopies were performed using standard gastroscopy Olympus equipment model 180. The data were collected using Endobase Software Olympus. The histology samples were collected using single use endoscopic forceps from Olympus. Histology examination were made using H + E method.

Prior the study, all participants (experimental and control group) filled the medical history survey. Moreover, during the whole experiment (i.e., at 0, 2, 4 week) subjects completed a nutritional interview and reported any symptoms while the application of honey.

This research is a pilot study before the administration of Manuka honey to populations of patients with incident GERD or gastritis.

### 2.4 Statistical analysis

The interpretation of the results was performed with MS Excel 2019 Analysis ToolPak software, one‐way analysis of variance (ANOVA) using the Tukey's post‐hoc test: different letters in the same row or column indicate statistical significance (at least *p* < .05).

## 3 RESULTS AND DISCUSSION

### 3.1 Impact of Manuka honey on GERD symptoms

The results of the initial survey showed that the common symptoms reported by patients were: heartburn (76.7%) and regurgitation (66.7%), regardless of the group (Manuka and Placebo). These symptoms are typical and confirmed by literature data (De Giorgi et al., 2006; Dent et al., 2005). Vakil et al. (2006) reported that heartburn and regurgitation concerns 75%–98% and 48%–91% of patients with GERD, respectively. In our study, other symptoms reported by patients, such as hoarseness, cough, chest pain, and so forth occurred in the range 16.7%–50% (Table 1).

**TABLE 1:** Initial symptoms reported by patients.

Group	Symptoms									
	Heartburn	Regurgitation	Throat pain	Esophagus pain	Hoarseness	Aphonia	Dyspnoea	Cough	Retrosternal chest pain	Sleep disturbance
Manuka	12	9	5	3	8	2	1	9	3	5
Placebo	11	11	4	4	7	3	4	2	7	4
Total (*n*) %	23^a^ 76.7%	20^b^ 66.7%	9^d^ 30.0%	7^d,e^ 23.3%	15^c^ 50%	5^e^ 16.7%	5^e^ 16.7%	11^d^ 36.7%	10^d^ 33.3%	9^d^ 30%

*Note*: The results were statistically analyzed by MS Excel 2019 Analysis ToolPak software. Different letters in the row “Total” indicate statistical significance (at *p* < .05).

Park et al. (2017) showed that typical symptoms (heartburn, regurgitation) were present in 98.4% of patients, while atypical symptoms were present in 65.6%. Most of our patients (80%) declared taking PPI and/or other drugs reducing the secretion of hydrochloric acid (H_2_‐blockers) (Table 2). Participants were not obliged to resign from taking medicaments during whole experiment. We decided to use Manuka honey in addition to main treatment of PPI in exchange for other typical treatment such as alkali or sucralfate. During the experiment (i.e., at 2 and 4 week) changes of symptoms in subjective assessment of patients were evaluated (Table 2). In the Manuka group, the improvement rate after 2 weeks was 86.7%, while in the Placebo group it was only 26.7%. At the end of the experiment (i.e., after 4 weeks) it was 100% and 40% in Manuka group and Placebo group, respectively. Great improvement (++) was reported by 73.3% of participants in Manuka group and only 20% in the Placebo group. It is worth noticing, that in sub‐group declaring non‐medication (*n* = 6), all subjects consuming Manuka honey (*n* = 4) reported improvement in subjective assessment, however this effect was not observed in Placebo group. Detailed data are presented in Table 2.

**TABLE 2:** Changes of subjective feelings of patients.

Manuka group				Placebo group			
Person	Drugs (yes/no)	2 weeks	4 weeks	Person	Drugs (yes/no)	2 weeks	4 weeks
1	No	+	+	1	Yes	0	0
2	Yes	++	++	2	Yes	0	+
3	Yes	++	++	3	No	0	0
4	Yes	++	++	4	Yes	0	−
5	Yes	++	++	5	Yes	0	0
6	No	++	++	6	Yes	+	++
7	No	++	++	7	Yes	0	++
8	No	++	++	8	Yes	+	++
9	Yes	0	+	9	Yes	0	0
10	Yes	+	+	10	Yes	+	+
11	Yes	+	++	11	Yes	+	+
12	Yes	++	++	12	Yes	0	0
13	Yes	++	++	13	No	0	0
14	Yes	+	+	14	Yes	0	0
15	Yes	0	++	15	Yes	0	0

*Note*: ++, great improvement; +, small improvement; 0, no effect; −, deterioration.

### 3.2 Endoscopy and histopatology

Besides patients' subjective assessments, the impact of Manuka honey has been evaluated by endoscopical examination. In the Manuka group, the improvement was stated for 73.3% of patients (Table 3), regardless of the diagnosis. In the sub‐groups of esophagitis A (*n* = 11) and esophagitis B (*n* = 4), the improvement rate was 81.8% and 50%, respectively. However, in the placebo group (Table 4), the general improvement rate was 33.3% (all cases were esophagitis A, *n* = 15).

**TABLE 3:** Results of endoscopy examination in Manuka group (before and after experiment).

Person	Drugs (yes/no)	Before	After	Evaluation
1	No	Esophagitis A	Normal	+
2	Yes	Esophagitis A, hiatal hernia	Hernia hiatus esophagi	+
3	Yes	Esophagitis A	Insufficientia cardiae	+
4	Yes	Esophagitis A	Esophagitis A	0
5	Yes	Esophagitis A	Normal	+
6	No	Esophagitis B	Normal	+
7	No	Esophagitis A, insufficientia cardiae	Normal	+
8	No	Esophagitis B	MUV	−
9	Yes	Esophagitis B	Esophagitis B	0
10	Yes	Esophagitis A	Gastritis antralis	+
11	Yes	Esophagitis A, hiatal hernia	Esophagitis A	0
12	Yes	Esophagitis A	Normal	+
13	Yes	Esophagitis A	Normal	+
14	Yes	Esophagitis B	Esophagitis A, gastritis antralis	+
15	Yes	Esophagitis A	Normal	+

*Note*: Esophagitis (A–D)—Los Angeles classification. +, improvement; 0, no effect; −, deterioration.

**TABLE 4:** Results of endoscopy examination in the placebo group (before and after experiment).

Person	Drugs (yes/no)	Before	After	Evaluation
1	Yes	Esophagitis A	Esophagitis A	0
2	Yes	Esophagitis A	Normal	+
3	No	Esophagitis A	Insufficientia cardiae	+
4	Yes	Esophagitis A	Insufficientia cardiae	+
5	Yes	Esophagitis A	Esophagitis A	0
6	Yes	Esophagitis A	Normal	+
7	Yes	Esophagitis A	Esophagitis A	0
8	Yes	Esophagitis A	Esophagitis B	−
9	Yes	Esophagitis A	Esophagitis A	0
10	Yes	Esophagitis A	Esophagitis A	0
11	Yes	Esophagitis A	Esophagitis B	−
12	Yes	Esophagitis A	Esophagitis A	0
13	No	Esophagitis A	Normal	+
14	Yes	Esophagitis A	Esophagitis A	0
15	Yes	Esophagitis A	Esophagitis A	0

*Note*: Esophagitis (A–D)—Los Angeles classification. +, improvement; 0, no effect; −, deterioration.

The results of the endoscopy have been confirmed in the histopathological examination of tissues collected from esophagus of patients. Initial results (at 0 week) showed that normal epithelium or erosive epithelium with inflammation were most common recognized both in Manuka and placebo groups. After 4 weeks of consuming Manuka honey, improvement was observed in 73.3% of subjects (epithelium without inflammation). However, in the placebo group the improvement rate was only 20%. Moreover, it is worth noticing, that in sub‐group declaring non‐medication, the histopatological examination showed the improvement only for patients consuming Manuka honey.

According to our research results, it should be underlined that consumption of Manuka Honey contributed to reducing the symptoms of GERD both in subjective assessment of patients and in endoscopical and histopathological examination. The benefits of Manuka honey may be due to the presence of bioactive compounds of antioxidant, anti‐inflammatory and bacteriostatic properties, in particular methylgloxal (MGO), hydrogen peroxide, polyphenols (Kwakman et al., 2011; Marshall et al., 2014; Mavric et al., 2008) and other unidentified compounds (Kato et al., 2012). Additional factors are high density, high viscosity, and low surface tension of honey, and therefore, it can stay longer in the esophagus as a coating on the mucosa (Lusby et al., 2005; Math et al., 2013).

### 3.3 Evaluation of subjects diet

During the study (i.e., at 0, 2 and 4 week) subjects completed questionnaires concerning their diet and smoking (Table 5). Obtained data were analyzed in particular for the presence of products that are associated with development of reflux, such as: fatty foods, chocolate, citrus fruits and juices, allium vegetables, spicy foods, carbonated drinks, coffee, and alcohol (Jarosz & Taraszewska, 2014; Meining & Classen, 2000; Oliver et al., 2010). Evaluation of our results showed the highest intake of fatty foods (86.7%), especially cheese, cream cheese and fatty fishes. On the other hand, low intake of fatty meat was observed, because subjects declared generally consumption of poultry. Second trigger factor of GERD was chocolate (73.3%), followed by sweets (66.7%). Some people declared excessive consumption of coffee and strong black tea—33.3% and 26.7% respectively. Among them were individuals who consumed even 4–6 cups of brewed coffee per day (*n* = 3). Less important in subjects diets was the intake of legumes, salty snacks, and alcohol (Table 5). Other researchers have also reported similar trigger factors of GERD. Oliver et al. reported especially the intake of fatty foods, alcohol, and chocolate (Oliver et al., 2010), whereas Jarosz and Taraszewska (2014) indicated fatty products, sweets and spicy products. Shapiro et al. (2007) showed that cholesterol, saturated fatty acids and percentage calories from fat are important agents. Moreover, in another study, products such as noodles, spicy foods, fatty meals, sweets, and alcohol were indicated (Song et al., 2011).

**TABLE 5:** Trigger factors of GERD (*n* = 30).

Trigger factor	Manuka group	Placebo group	Total	%
Fatty foods	15	11	26	86.7^a^
Chocolate	12	10	22	73.3^b^
Sweets	11	9	20	66.7^b^
Coffee*	4	6	10	33.3^c^
Legumes	5	4	9	30.0^c^
Strong black tea*	3	5	8	26.7^c^
Salty snacks	4	2	6	20.0^d^
Alcohol	2	3	5	16.7^d^
Smoking	3	2	5	16.7^d^
Citrus fruits and juices	2	2	4	13.3^e^
Carbonated drinks	1	2	3	10.0^e^

*Note*: The results were statistically analyzed by MS Excel 2019 Analysis ToolPak software. Different letters in the last column indicate statistical significance (at *p* < .05).

* 3 and more cups per day.

Taking into account the nutritional guidelines for GERD, it was found that diet of 90% of patients were incorrect. After filling the initial questionnaire, subjects got information about gaps in their nutrition. Despite this, after 4 weeks a large number of people still had inappropriate diet (66.7%, *n* = 20), including 80% (*n* = 12) in the Manuka group. It is worth noticing, that eight patients consuming Manuka honey had determined the improvement of endoscopical and histopathological results in spite of not changing the diet.

To the best of authors knowledge, there is no other study concerning the use of Manuka honey in GERD. Some studies show the impact of various food products in esophagitis and dyspepsia of laboratory animals or humans. Giri et al. (2015) in the experiment conducted on rats concluded that lycopene may have a protective effect against esophagitis due to scavenging of reactive oxygen species and anti‐inflammatory potential. Mohtashami et al. (2015) showed that supplementation of traditional honey based formulation of *Nigella sativa* (5 mL *N. sativa* oil orally daily) can improve the control of the symptoms of patients with functional dyspepsia. It also significantly improves the rate of *Helicobacter pylori* eradication and quality of life in these patients. Some of publications refer to the effectiveness of Manuka honey against *H. pylori* (Al‐Somal et al., 1994; Alvarez‐Suarez et al., 2010; Mc Govern et al., 1999; Nzeako & Al‐Namaani, 2006) and other pathogens of the gastrointestinal tract (Lin et al., 2011). Lin et al. (2011) showed that Manuka honey is significantly more effective against most gastrointestinal bacteria than artificial honey.

According to our research results, the application of Manuka honey may improve treatment of gastro‐esophageal reflux disease, but this requires confirmation by further studies on a larger group of patients.

## 4 CONCLUSIONS

Manuka honey seems to be effective in GERD, which have been confirmed by subjective feelings of patients and by endoscopic and histopathologic examination. We concluded that consumption of Manuka honey combined with proper diet and drugs (proton‐pump inhibitors) could be a triple therapy regimen for GERD. Our research is a pilot study before administration of Manuka honey to populations of patients with GERD. The results are promising and may facilitate the quality of life of patients with reflux disease, for example relieving heartburn and regurgitation.

## AUTHOR CONTRIBUTIONS

**Michał Gośliński:** Conceptualization (equal); data curation (equal); formal analysis (equal); investigation (equal); methodology (equal); writing – original draft (equal). **Dariusz Nowak:** Data curation (equal); investigation (equal); methodology (equal); writing – original draft (equal). **Roman Mindykowski:** Investigation (equal). **Wojciech Kulewski:** Investigation (equal). **Cezary Popławski:** Conceptualization (equal); investigation (equal); methodology (equal); supervision (equal).

## CONFLICT OF INTEREST STATEMENT

The authors declare that there are no conflict of interest.

## Data Availability

The data that support the findings of this study are available on request from the corresponding author.
